# Intensity-Modulated Radiotherapy and Three-Dimensional Conformal Radiotherapy Combined with Intracavitary Posterior Radiotherapy for the Treatment of Medium-Term and Advanced Cervical Cancer: Efficacy, Safety and Prognostic Factors

**DOI:** 10.3389/fsurg.2022.906117

**Published:** 2022-05-23

**Authors:** Kewen Yu, Liping Zhou

**Affiliations:** ^1^ Department of gynecology, Ningbo Women and Children's Hospital, Ningbo, China; ^2^ Department of gynecology, Zhuji People’s Hospital of Zhejiang Province, Zhuji, China

**Keywords:** medium-term and advanced cervical cancer, intensity modulated radiation therapy, three-dimensional conformal radiotherapy, efficacy, prognosis

## Abstract

**Objective:**

To explore the efficacy, safety, and prognostic factors of intensity modulated radiation therapy (IMRT) and three dimensional conformal radiation therapy (3D-CRT) combined with intracavitary posterior radiotherapy for medium-term and advanced cervical cancer.

**Methods:**

Retrospectively analyze the clinical data of 104 patients with medium-term and advanced cervical cancer who were treated in the radiotherapy department of our hospital from September 2015 to March 2017. According to the different radiotherapy techniques, they were divided into the IMRT combined with intracavitary posterior radiotherapy group (*n* = 52) and the 3D-CRT combined with intracavitary posterior radiotherapy group (*n* = 52). Observe and compare the short-term efficacy, occurrence of adverse reactions and overall survival rate of the two groups. The clinicopathological characteristics of the survival group and the death group were compared, and univariate analysis and multiple logistic regression models were used to analyze the relationship between the clinicopathological characteristics and the patient’s prognosis.

**Results:**

The total effective rate of IMRT combined with intracavitary posterior radiotherapy group was 96.15%, which was higher than that of 3D-CRT combined with intracavitary posterior radiotherapy group (88.46%), but the difference was not statistically significant (*p *> 0.05). The incidence of digestive system injury, thrombocytopenia, and radiation proctitis in the IMRT combined intracavitary posterior radiotherapy group was lower than that of the 3D-CRT combined intracavitary posterior radiotherapy group, and the differences were statistically significant (*p *< 0.05). The prognosis and survival of the two groups of patients were similar, and the difference was not statistically significant (*p *> 0.05). Pathological classification, clinical stage, and lymph node metastasis are independent influencing factors of 3-year prognosis in patients with medium-term and advanced cervical cancer (*p *< 0.05).

**Conclusion:**

IMRT combined with intracavitary posterior radiotherapy is equivalent to 3D-CRT combined with intracavitary posterior radiotherapy, but it can reduce the incidence of adverse reactions in patients with medium-term and advanced cervical cancer, and has higher safety. Pathological typing, clinical staging, Lymph node metastasis were independent factor affecting the prognosis of patients. In clinical treatment, IMRT combined with intracavitary posterior radiotherapy is more recommended as a treatment plan for patients with medium-term and advanced cervical cancer.

## Introduction

Cervical cancer is one of the most common malignant tumors in women. Early stage cervical cancer is usually treated with surgery, while radiotherapy and chemotherapy are the main treatments in the medium-term and advanced stages (1). At present, external irradiation combined with intracavitary posterior radiotherapy and concurrent chemotherapy is the standard method for the treatment of medium-term and advanced cervical cancer. Traditional radiotherapy methods such as four-field box-type and front-to-back penetrating irradiation have been used earlier and are widely used, but their damage to the digestive tract, urinary tract and hematopoietic systems is relatively serious (2, 3). Intensity modulated radiation therapy (IMRT) and three dimensional conformal radiation therapy (3D-CRT) are new radiotherapy techniques, and there is no unified understanding on the specific effects of these two techniques. However, studies have confirmed that these two techniques can increase the irradiation dose of target area and effectively reduce the irradiation dose of surrounding normal tissues and organs, effectively control tumors and reduce damage to the body (4, 5). Whether IMRT and 3D-CRT can replace traditional radiotherapy in the treatment of medium-term and advanced cervical cancer is worth looking forward to. Therefore, in this study, IMRT combined with intracavitary radiotherapy was used to treat patients with medium-term and advanced cervical cancer, and compared with 3D-CRT combined with intracavitary posterior radiotherapy, in order to provide theoretical support for the efficient treatment of patients with medium-term and advanced cervical cancer. The specific research is shown as follows.

## Materials and Methods

### General Information

A total of 104 patients with medium-term and advanced cervical cancer who were admitted to our hospital from September 2015 to March 2017 were selected. All patients met the diagnostic criteria for medium-term and advanced cervical cancer (6); Diagnosed by pathological and imaging examinations; tumor FIGO stage was IIB–IVA; Bian’s score was ≥70 points; expected survival >3 months; abnormal liver and kidney function and other organ diseases were excluded. According to the treatment method, the patients were divided into IMRT and 3D-CRT combined with intracavitary posterior radiotherapy groups, with 52 patients in each group. The age of IMRT combined with intracavitary posterior radiotherapy group was 32–77 years old, with an average age of (55.03 ± 7.14) years; 32 cases of IIB, 10 cases of IIIA, 9 cases of IIIB, and 1 case of IVA; 49 cases of squamous cell carcinoma and 3 cases of adenocarcinoma; 30 patients had tumor diameter ≥4 cm, and 22 patients had tumor diameter <4 cm. The age of 3D-CRT combined with intracavitary posterior radiotherapy group was 33–78 years old, with an average of (54.86 ± 7.25) years old; 34 cases of IIB, 9 cases of IIIA, 8 cases of IIIB, and 1 case of IVA; 50 cases of squamous cell carcinoma and 2 cases of adenocarcinoma; 29 patients had tumor diameter ≥4 cm, and 23 patients had tumor diameter <4 cm. There was no statistical difference in general clinical data such as age, FIGO stage, pathological classification and tumor diameter between the two groups (*p* > 0.05), which were comparable.

### Research Methods

In terms of the IMRT combined with intracavitary posterior radiotherapy group: One day before localization, the patients underwent bowel preparation by oral administration of Meglumine. Before localization, the patients’ bladder was filled and the rectum was emptied. The joints were fixed with vacuum pad and thermoplastic film, and CT scan was performed. Clinical target volume (CTV): including the primary area of cervical tumor (parametrial triangle, cervix, vagina, etc.) and pelvic metastatic area (parametrium, paravaginal tissue, pelvic lymphatic area, etc.). Planned target volume (PTV): CTV was expanded by 5 mm in the S/I, A/P, and R/L directions, respectively. Gross tumor target volume (GTV): the cervical mass and lymph node metastases that have been diagnosed by imaging or definite diagnosis. Lymph node area delineation: The lymph node area and corresponding blood vessel area were delineated along the lymph node area and the corresponding blood vessel area in patients diagnosed with lymph node metastasis, followed by delineation of the para-aortic lymph nodes, anterior iliac, external iliac, internal iliac, common iliac and obturator foramen. The range was from the fifth lumbar vertebra to the obturator foramen. The PTV dose was 1.8 Gy/time, and the total dose was 48.60–50.40 Gy/27–28, 5 times a week. PTV adopted 7-field intensity-modulated irradiation, and was irradiated with an isodose curve of 95% of PTV, and the minimum and maximum doses of the target area were within the range of ±10% of the prescribed dose. After 25 times of external irradiation, the irradiation was stopped, intracavitary posterior radiotherapy was performed once a week, 6–7 Gy/time, with a total dose of 30–36 Gy/5–6 f. The radiation doses of the small intestine, rectum and bladder were V45 < 20%, V45 < 40% and V45 < 30%, respectively.

In terms of the 3D-CRT combined with intracavitary posterior radiotherapy group: the positioning and target delineation methods were the same as above. The target area was irradiated by four-field box type, 6MV-X-ray, 180–200 cGy/time, the total dose was 4,500–5,000 cGy. Conventional segmentation irradiation, 5 times a week. The method of intracavitary posterior radiotherapy was the same as above.

Observation indicators: The short-term efficacy and adverse reactions of the two groups of patients were recorded, analyzed and compared, and the adverse reactions included acute and chronic radiation injury. Acute and chronic radiation injuries were divided into grades I, II, III, and IV. The higher the grade, the more severe the adverse reaction. Acute radiation injury included digestive tract, blood, and urinary system injury; chronic radiation injury was mainly intestinal and urinary system injury, including radiation cystitis and proctitis. After radiotherapy, consolidation chemotherapy was performed according to the specific conditions of the patients. During hospitalization, the patients were regularly checked for blood routine every week. In the event of an acute chemoradiotherapy reaction, medical staff need to timely treat the symptoms according to the specific conditions of the patient.

Efficacy evaluation: The changes in tumor size were observed by CT or MRI, and graded according to the WHO efficacy evaluation criteria. including disease progression (PD): the product of the largest vertical diameter and the largest diameter of the lesion (the product of the two diameters) increased by more than 25% compared with that before radiotherapy, and time continued more than 30 days; stable disease (SD): the two-dimensional product of the lesion decreased by less than 50% or increased by less than 25% compared with that before radiotherapy, and lasted for more than 30 days; partial remission (PR): the two-dimensional product of the lesion decreased by at least 50% compared with that before radiotherapy, duration of more than 30 days; complete remission (CR): the lesions completely disappeared, the duration of more than 30 days. Total effective rate = (PR + CR) / total number of cases × 100%.

Follow-up: Patients were regularly followed up every 3 months by outpatient or telephone, all patients were followed up for 3 years. The follow-up contents included patient survival time and adverse reactions. Survival time was calculated from the time of diagnosis to the date of death or the end of follow-up.

### Statistical Methods

SPSS 22.0 software was used for processing. The measurement data conforming to normal distribution were expressed as mean ± standard deviation, and t test was used for comparison. Count data were expressed as (%), and χ^2^ test was used for comparison. Survival curves were drawn by using the Kaplan-Meier method. The test level was α = 0.05, and *p* < 0.05 was considered statistically significant.

## Results

### Comparison of Short-Term Curative Effect between Two Groups of Patients

The results showed that the total effective rate of IMRT combined with intracavitary posterior radiotherapy group was 96.15%, which was higher than that of 3D-CRT combined with intracavitary posterior radiotherapy group (88.46%), but the difference was not statistically significant (*p* > 0.05), as shown in Table 1.

**Table 1:** Comparison of effective rates between the two groups of patients (*n*, %).

Group	PD	SD	PR	CR	PR + CR
IMRT combined with intracavitary posterior radiotherapy group	0	2	27	23	50 (96.15)
3D-CRT combined with intracavitary posterior radiotherapy group	0	5	26	20	46 (88.46)
*χ^2^*	–	–	–	–	2.167
*p*	–	–	–	–	0.141

### Comparison of Adverse Reactions of the Two Groups of Patients

The results showed that the incidence of digestive system injury, thrombocytopenia, and radiation proctitis in the IMRT combined with intracavitary posterior radiotherapy group were 28.85%, 9.62%, and 19.23%, respectively, which were lower than those in the 3D-CRT combined with intracavitary posterior radiotherapy group (55.77%, 40.38%, 48.08%), the differences were statistically significant (*p* < 0.05). There was no significant difference in the incidence of hemoglobin reduction, leukopenia, urinary system injury and radiation cystitis between the two groups (*p* > 0.05), as shown in Table 2.

**Table 2:** Comparison of the occurrence of adverse reactions of the two groups of patients (*n*, %).

Classification	Adverse reaction	IMRT combined with intracavitary posterior radiotherapy group	3D-CRT combined with intracavitary posterior radiotherapy group	*χ* ^2^	*p*
Acute	Digestive damage	15 (28.85)	29 (55.77)	7.721	0.005
	Thrombocytopenia	5 (9.62)	21 (40.38)	13.128	0.001
	Decreased hemoglobin	24 (46.15)	26 (50.00)	0.154	0.695
	Leukopenia	35 (67.31)	34 (65.38)	0.043	0.836
	Urinary system damage	8 (15.38)	11 (21.15)	0.580	0.446
Chronic	Radiation proctitis	10 (19.23)	25 (48.08)	9.689	0.002
	Radiation cystitis	3 (5.77)	6 (11.54)	1.095	0.295

### Comparison of Prognosis and Survival between the Two Groups of Patients

**Figure 1:**
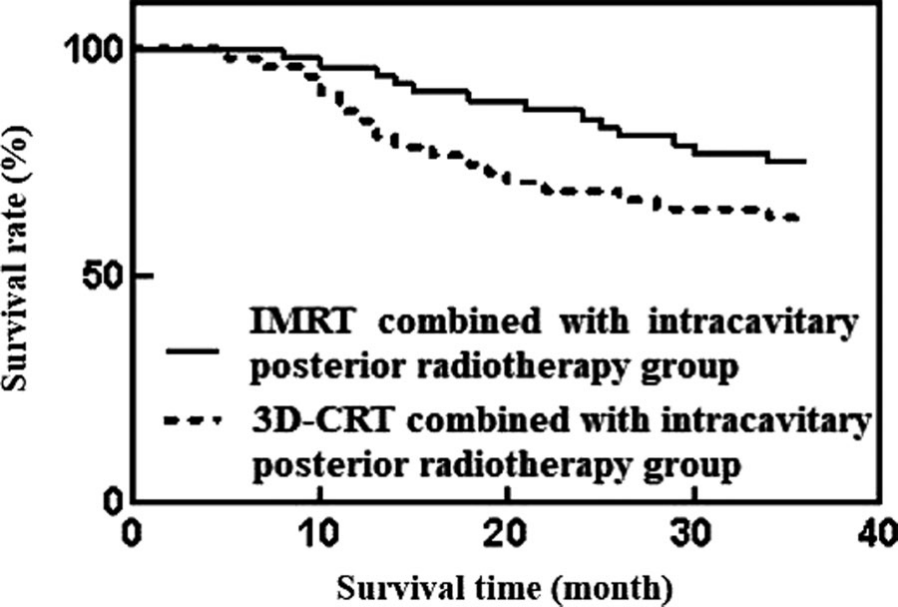
Comparison of prognosis and survival between the two groups of patients.

The results showed that during the 3-year follow-up, the total number of deaths was 32 cases. Among them, the mortality rate of IMRT combined with intracavitary posterior radiotherapy was 25.00% (13/52) lower than that of 3D-CRT combined with intracavitary posterior radiotherapy group, which was 36.54% (19/52), and the median survival time of both groups was 36 months. The difference was not statistically significant (*p* > 0.05), as shown in Figure 1.

### Analysis of Prognostic Factors of Patients with Medium-Term and Advanced Cervical Cancer

The results showed that there were significant differences in case type, clinical stage, tumor diameter, and lymph node metastasis between the survival group and the death group (*p* < 0.05). There was no statistically significant difference in age (*p* > 0.05) as shown in Table 3.

**Table 3:** Analysis of prognostic factors of patients with medium-term and advanced cervical cancer (*n*, %).

Influencing factors	Survival group (*n* = 72)	Death group (*n* = 32)	*χ* ^2^	*p*
Age (Years)				
≥55	35 (48.61)	14 (43.75)	0.210	0.647
<55	37 (51.39)	18 (56.25)		
Pathological typing				
Squamous cell carcinoma	71 (98.61)	28 (87.50)	5.976	0.015
Adenocarcinoma	1 (1.39)	4 (12.50)		
Clinical stage				
II B	41 (56.94)	25 (78.12)	4.286	0.038
III–IV	31 (43.06)	7 (21.88)		
Tumor diameter (cm)				
≥4	46 (63.89)	13 (40.62)	4.85	0.027
<4	26 (36.11)	19 (59.38)		
Lymph node metastasis				
Yes	32 (44.44)	26 (81.25)	12.166	0.001
No	40 (55.56)	6 (18.75)		

### Multivariate Analysis of Prognosis of Patients with Medium-Term and Advanced Cervical Cancer

Logistic regression analysis showed that pathological type, clinical stage and lymph node metastasis were independent influencing factors of 3-year prognosis of patients with medium-term and advanced cervical cancer (*p* < 0.05), while tumor diameter had no significant effect on 3-year prognosis of patients (*p* > 0.05), as shown in Tables 4 and 5.

**Table 4:** Assignment table.

Influencing factors	Assignment
Pathological typing	Squamous cell carcinoma = 1, Adenocarcinoma = 2
Clinical stage	II B = 1, III ∼ IV = 2
Tumor diameter	≥4 cm = 1, <4 cm = 2
Lymph node metastasis	Yes = 1, No = 2

**Table 5:** Multivariate analysis of prognosis of patients with medium-term and advanced cervical cancer.

Influencing factors	*B*	*SE*	*Walds*	df	Sig.	Exp(B)
Pathological typing	2.615	1.034	6.398	1	0.027	5.492
Clinical stage	1.569	0.523	8.741	1	0.015	6.937
Tumor diameter	1.037	0.594	3.317	1	0.0145	2.459
Lymph node metastasis	1.375	0.509	7.321	1	0.023	6.558

## Discussion

The incidence of cervical cancer is increasing year by year, and the patients tend to be younger. Therefore, it is particularly important to reduce the mortality rate of cervical cancer and control the development of cervical cancer (7). External beam radiation combined with intracavitary radiotherapy and concurrent chemotherapy in the treatment of medium-term and advanced cervical cancer can prevent tumor metastasis and local recurrence. The scope of external irradiation not only includes the uterus, paracyngeal tissues, cervix and vagina, but also needs to cover the pelvic lymphatic drainage area (8, 9). Traditional external irradiation techniques are relatively backward, and the incidence of various complications has increased, which has been gradually replaced by IMRT, 3D-CRT and other new radiotherapy technologies (10).

3D-CRT is based on the reconstruction of three-dimensional image of human body structure, and can accurately distinguish normal tissue and tumor tissue. The radiation dose of the rectum, bladder and other organs at risk can be reduced by adjusting the radiation direction and changing the radiation dose (11). IMRT is an in vitro three-dimensional irradiation method developed through improved 3D-CRT technology. By adjusting the output dose, the radiation shape in all three-dimensional directions can be kept consistent with the target area, and the radiation dose of organs at risk and normal tissues around the target area can be reduced. so as to effectively treat and control tumors (12). Compared with 3D-CRT technology, IMRT technology can better adapt to irregularly shaped tumors, and at the same time, by controlling the dose intensity, a high-dose target area can be concentrated in the target area (13). IMRT can reduce the acute myelosuppression and other reactions caused by concurrent chemotherapy, which can reduce the harm to surrounding tissues and reduce the occurrence of various complications.

IMRT and 3D-CRT are widely used in cancer treatment. Guillemin et al. (14) used IMRT and 3D-CRT in the treatment of non-small cell lung cancer and found that IMRT can more effectively reduce the radiation dose to the surrounding organs at risk of patients. In cervical cancer, Contreras’ team (15) found that IMRT can reduce the amount of rectal and bladder tissue around the tumor, ensure the coverage of tumor tissue, and alleviate complications caused by radiotherapy. This study found that the total effective rate of IMRT combined with intracavitary posterior radiotherapy was higher than that of 3D-CRT combined with intracavitary posterior radiotherapy, but the difference was not statistically significant. There was no significant difference in the overall survival rate and median survival time between the two groups. The results showed that the treatment efficacy of the two regimens were equivalent. However, the incidences of gastrointestinal tract injury, thrombocytopenia and subsequent radiation proctitis in the IMRT combined with intracavitary posterior radiotherapy group were significantly lower than those in the 3D-CRT combined with intracavitary posterior radiotherapy group. This suggested that when all conditions of intracavitary posterior radiotherapy were the same, IMRT can relieve gastrointestinal and hematological injury, with higher safety.

The results of this study showed that pathological type, clinical stage and lymph node metastasis were independent prognostic factors for patients with medium-term and advanced cervical cancer. Among them, patients with squamous cell carcinoma, late clinical stage, and lymph node metastasis have higher mortality rates. This may be related to the fact that the cancer cells of squamous cell carcinoma patients have invaded into the muscle layer, the distance from the radiation source is far, patients with advanced staging have increased tumor uncontrollability, and patients with lymph node metastasis have wider tumor distribution (16, 17). Yüksel et al. (18), Ramlov et al. (19) found that case typing, tumor size, lymph node metastasis, etc. are all risk factors for the prognosis of patients with medium-term and advanced cervical cancer. However, this study showed that tumor size was not a prognostic factor for patients with medium-term and advanced cervical cancer, which was partially deviated from previous studies. This may be related to the small sample size and short follow-up time in this study. It is necessary to expand the sample size and extend the follow-up time in the future to improve the credibility of the research results.

In conclusion, IMRT combined with intracavitary posterior radiotherapy has the same curative effect as 3D-CRT combined with intracavitary posterior radiotherapy, but it can reduce the incidence of adverse reactions of patients with medium-term and advanced cervical cancer and has higher safety. Pathological type, clinical stage, and lymph node metastasis are independent factors affecting the prognosis of patients. In clinical treatment, IMRT combined with intracavitary posterior radiotherapy is more recommended as the treatment plan for patients with medium-term and advanced cervical cancer.

## Data Availability

The original contributions presented in the study are included in the article/Supplementary Material, further inquiries can be directed to the corresponding author/s.

## References

[1] Williamson CW Liu HC Mayadev J Mell LK. Advances in external beam radiation therapy and brachytherapy for cervical cancer. Clin Oncol (R Coll Radiol). (2021) 33:567–78. 10.1016/j.clon.2021.06.012 34266728

[2] Lee J Lin JB Chang CL Sun FJ Wu MH Jan YT Impact of para-aortic recurrence risk-guided intensity-modulated radiotherapy in locally advanced cervical cancer with positive pelvic lymph nodes. Gynecol Oncol. (2018) 148:291–8. 10.1016/j.ygyno.2017.12.003 29269219

[3] Ping Q Zeng J Sun P Qu P Jiang S Hu Y. Efficacy of preoperative brachytherapy for controlling vaginal bleeding in early-stage cervical cancer: a retrospective study. Transl Cancer Res. (2021) 10:3259–67. 10.21037/tcr-21-467 35116632 PMC8798223

[4] Yang H Feng C Cai BN Yang J Liu HX Ma L. Comparison of three-dimensional conformal radiation therapy, intensity-modulated radiation therapy, and volumetric-modulated arc therapy in the treatment of cervical esophageal carcinoma. Dis Esophagus. (2017) 30:1–8. 10.1111/dote.12497 27629865

[5] Arul Ponni TR Avinash HU Nirmala S Janaki MG Kirthi Koushik AS. Optimal technique of radiotherapy for carcinoma cervix in developing countries: dosimetric and logistic comparison. J Cancer Res Ther. (2018) 14:1207–13. 10.4103/jcrt.JCRT_454_17 30488831

[6] Massad LS Einstein MH Huh WK Katki HA Kinney WK Schiffman M 2012 updated consensus guidelines for the management of abnormal cervical cancer screening tests and cancer precursors. Obstet Gynecol. (2013) 121:829–46. 10.1097/AOG.0b013e3182883a34 23635684

[7] Aghili M Andalib B Karimi Moghaddam Z Maddah Safaie A Amoozgar Hashemi F Mousavi Darzikolaie N. Concurrent chemo- radiobrachytherapy with cisplatin and medium dose rate intra- cavitary brachytherapy for locally advanced uterine cervical cancer. Asian Pac J Cancer Prev. (2018) 19:2745–50. 10.22034/APJCP.2018.19.10.2745 30360600 PMC6291044

[8] Yu H Zhang L Li D Liu N Yin Y Zhang L Postoperative adjuvant chemotherapy combined with intracavitary brachytherapy achieved the equivalent survival compared with concurrent chemoradiotherapy in cervical cancer patients with intermediate-risk. Jpn J Clin Oncol. (2019) 49:714–8. 10.1093/jjco/hyz057 31329905

[9] Xiang J Liu F Wang B Chen L Liu W Tan S. A literature review on maillard reaction based on milk proteins and carbohydrates in food and pharmaceutical products: advantages, disadvantages, and avoidance strategies. Foods. (2021) 10:1998. 10.3390/foods10091998 34574107 PMC8472807

[10] Mohanty SK Chopra S Mudaliar A Kannan S Mahantshetty U Engineer R A comparative analysis of quality of life after postoperative intensity-modulated radiotherapy or three-dimensional conformal radiotherapy for cervical cancer. Indian J Cancer. (2018) 55:327–35. 10.4103/ijc.IJC_453_17 30829265

[11] Dracham CB Mahajan R Rai B Elangovan A Bhattacharya T Ghoshal S. Toxicity and clinical outcomes with definitive three-dimensional conformal radiotherapy (3DCRT) and concurrent cisplatin chemotherapy in locally advanced cervical carcinoma. Jpn J Clin Oncol. (2019) 49:146–52. 10.1093/jjco/hyy164 30452664

[12] Shang H Pu Y Wang W Dai Z Jin F. Evaluation of plan quality and robustness of IMPT and helical IMRT for cervical cancer. Radiat Oncol. (2020) 15:34. 10.1186/s13014-020-1483-x. 32054496 PMC7020599

[13] Lin Y Chen K Lu Z Zhao L Tao Y Ouyang Y Intensity-modulated radiation therapy for definitive treatment of cervical cancer: a meta-analysis. Radiat Oncol. (2018) 13:177. 10.1186/s13014-018-1126-7 30217165 PMC6137729

[14] Guillemin F Berger L Lapeyre M Bellière-Calandry A. Dosimetric and toxicity comparison of IMRT and 3D-CRT of non-small cell lung cancer. Cancer Radiother. (2021) 25:747–54. French. 10.1016/j.canrad.2021.03.001 34183268

[15] Contreras J Srivastava A Chundury A Schwarz JK Markovina S Thaker PH Long-term outcomes of intensity-modulated radiation therapy (IMRT) and high dose rate brachytherapy as adjuvant therapy after radical hysterectomy for cervical cancer. Int J Gynecol Cancer. (2020) 30:1157–61. 10.1136/ijgc-2020-001412 32527770

[16] Xu XH Chen YC Xu YL Feng ZL Liu QY Guo X Garcinone E blocks autophagy through lysosomal functional destruction in ovarian cancer cells. World J Tradit Chin Med. (2021) 7:209–16. 10.4103/wjtcm.wjtcm_83_20

[17] Aslan K Haberal A Akıllı H Meydanli MM Ayhan A. Prognostic value of the number of the metastatic lymph nodes in locally early-stage cervical cancer: squamous cell carcinoma versus non-squamous cell carcinoma. Arch Gynecol Obstet. (2021) 304:1279–89. 10.1007/s00404-021-06030-w 33772630

[18] Yüksel D Karataş Şahin E Ünsal M Çakır C Kılıç Ç Kimyon Cömert G The prognostic factors in 384 patients with FIGO 2014 stage IB cervical cancer: what is the role of tumor size on prognosis? Eur J Obstet Gynecol Reprod Biol. (2021) 266:126–32. 10.1016/j.ejogrb.2021.09.028 34634671

[19] Ramlov A Pedersen EM Røhl L Worm E Fokdal L Lindegaard JC Risk factors for pelvic insufficiency fractures in locally advanced cervical cancer following intensity modulated radiation therapy. Int J Radiat Oncol Biol Phys. (2017) 97:1032–9. 10.1016/j.ijrobp.2017.01.026 28332986

